# Emergence of high-risk ST595 and ST640 clones of carbapenem-resistant *Serratia marcescens*: insights from genomic and virulence profiling during a nosocomial epidemic

**DOI:** 10.3389/fmicb.2025.1681911

**Published:** 2025-10-09

**Authors:** Yan Zhang, Tong Cao, Jie Zheng, Lingning Meng, Shuo Gao, Han Shen, Wanqing Zhou, Xiaoli Cao

**Affiliations:** ^1^Department of Clinical Laboratory, Nanjing Drum Tower Hospital, Affiliated Hospital of Medical School, Nanjing University, Nanjing, Jiangsu, China; ^2^Department of Clinical Laboratory, Children’s Hospital of Nanjing Medical University, Nanjing, Jiangsu, China

**Keywords:** *Serratia marcescens*, carbapenem resistance, virulence factors, whole-genome sequencing, sequence typing, biofilm formation, serum resistance, nosocomial outbreak

## Abstract

**Objective:**

Carbapenem-resistant *Serratia marcescens* (CRSM) poses a significant threat in hospital settings due to its potential for persistence and transmission. This study aims to elucidate the virulence landscape of carbapenem-resistant *Serratia marcescens* (CRSM) during a hospital outbreak and to identify high-risk clones with enhanced persistence and transmission potential.

**Methods:**

A total of 52 CRSM clinical isolates collected during a nosocomial outbreak were subjected to whole-genome sequencing (WGS) for comprehensive analysis of virulence factor (VF) profiles, sequence types (STs), and phylogenetic relationships. Based on genomic clustering, representative isolates were selected for *in vitro* phenotypic assays, including bacterial growth kinetics, biofilm formation, and serum bactericidal activity.

**Results:**

All the CRSM carried *bla*KPC-2 (52/52), other carbapenemases detected included *bla*NDM-1 (*n* = 3), *bla*OXA-23 (*n* = 6), and *bla*OXA-66 (*n* = 6). In addition, the majority of them harbored *bla*SRT-1 (48/52), *bla*CTX-M-14 (42/52) and *bla*TEM-1B (25/52). Two major epidemic clones were identified: ST595 (*n* = 25) and ST640 (*n* = 17), indicating clonal expansion during the outbreak. All isolates carried a conserved set of core VFs, including *shlA*, *shlB*, *hlyA*, and multiple genes associated with motility and iron acquisition. Additional accessory VFs, such as *plcN* (*n* = 33) and *fliF* (*n* = 20), were variably distributed. Phenotypic characterization revealed that specific isolates, notably SM60, simultaneously exhibited accelerated growth, robust biofilm formation, and marked serum resistance—a combination of traits that may drive both environmental persistence and immune evasion in hospital settings.

**Conclusion:**

This study provides the first integrated genomic and phenotypic characterization of CRSM during a documented hospital outbreak, highlighting ST595 and ST640 as high-risk clones with distinct virulence signatures. The convergence of rapid growth, biofilm capacity, and serum resistance in select isolates underscores their potential role in prolonged colonization and nosocomial spread. These findings emphasize the urgent need for genomic surveillance and targeted infection control strategies to curb the dissemination of emerging CRSM clones.

## 1 Introduction

*Serratia marcescens* is an opportunistic Gram-negative pathogen increasingly recognized as a cause of healthcare-associated infections, including bloodstream infections, pneumonia, urinary tract infections, and outbreaks in intensive care units (ICUs) (da Silva et al., 2021; Huang et al., 2021; Johnson et al., 2020; Kim et al., 2020; Muyldermans et al., 2021; Bechmann et al., 2023). In recent years, the emergence of carbapenem-resistant *S. marcescens* (CRSM) has raised significant concern worldwide due to limited therapeutic options and its ability to persist in hospital environments (Liu et al., 2025).

Although the molecular mechanisms underlying carbapenem resistance—such as the acquisition of metallo-*β*-lactamases (*bla*IMP*, bla*NDM), —have been partially characterized (Li et al., 2025), relatively little is known about the broader resistance gene landscape in *S. marcescens* and how it contributes to persistence, dissemination, and outbreaks (Tavares-Carreon et al., 2023). Recent studies have documented increasingly complex resistomes in clinical isolates, including *bla*KPC-2 and other determinants conferring resistance to aminoglycosides, quinolones, sulfonamides, and tetracyclines (Chen et al., 2025). These resistance repertoires may enhance the survival and spread of CRSM in hospital settings, particularly under selective antibiotic pressure.

In contrast to better-studied multidrug-resistant pathogens such as *Klebsiella pneumoniae* and *Acinetobacter baumannii*, the virulence determinants of *S. marcescens*—particularly in the context of carbapenem resistance—remain insufficiently explored. While several virulence factors (e.g., cytotoxins *shlA/B*, flagellar transcriptional regulator *fhlC*, siderophore-related and biofilm-associated genes) have been described in laboratory strains (Stella et al., 2021), their prevalence, clonal associations, and contribution to clinical CRSM infections are poorly defined. Moreover, there is a lack of integrative studies combining genomic and phenotypic data, leaving the relationships between resistance, virulence, and epidemiological success largely unexplored (Ono et al., 2022).

To address this knowledge gap, we investigated a collection of CRSM isolates obtained from Nanjing Drum Tower Hospital. Importantly, many of these isolates were associated with a nosocomial outbreak, suggesting the dissemination of high-risk epidemic clones (Aracil-Gisbert et al., 2024; Pérez-Viso et al., 2021). Rather than aiming to test a single mechanistic hypothesis, this study was designed as an exploratory baseline analysis to describe the genomic and phenotypic characteristics of these outbreak-associated isolates. Specifically, we applied whole-genome sequencing (WGS) alongside phenotypic assays—growth kinetics, biofilm formation, and serum resistance—chosen because these traits are relevant to bacterial fitness, immune evasion, and environmental persistence (Williams et al., 2022).

The objectives of this study were therefore to (i) define the resistance and virulence gene repertoires of CRSM isolates, (ii) delineate their clonal structure and phylogenetic relationships, and (iii) examine phenotypic traits linked to persistence and transmission. By focusing on outbreak-associated lineages ST595 and ST640, we aim to provide a systematic characterization of these epidemic clones, laying the groundwork for future hypothesis-driven studies into the mechanisms of resistance, virulence, and outbreak potential.

## 2 Materials and methods

### 2.1 Bacterial isolates

A total of 52 non-duplicate clinical isolates of CRSM were collected from Nanjing Drum Tower Hospital, the Affiliated Hospital of Nanjing University Medical School, during a documented nosocomial outbreak between June 2018 and September 2019. The isolates were recovered from multiple clinical specimens, including respiratory secretions (*n* = 47), blood (*n* = 3), hydrothorax (*n* = 1) and wound swabs (*n* = 1), representing both infection and colonization cases identified in intensive care units and general wards. All isolates were initially identified using the Vitek 2.0 Compact system (bioMérieux, France) and further confirmed by Matrix-Assisted Laser Desorption/Ionization Time-of-Flight Mass Spectrometry (MALDI-TOF MS; bioMérieux, France). Carbapenem resistance was determined by testing the minimum inhibitory concentrations (MICs) of imipenem and meropenem according to CLSI 2024 guidelines (CLSI, 2024). In addition, a broader panel of antibiotics—including cephalosporins, aminoglycosides, fluoroquinolones, and trimethoprim-sulfamethoxazole—was tested in parallel using the broth microdilution method, and results were interpreted according to CLSI 2024 breakpoints (Supplementary Table 1). These data provided a comprehensive resistance profile to support genomic findings.

### 2.2 Whole-genome sequencing

Genomic DNA of the 52 CRSM isolates was extracted using a bacterial genomic DNA extraction kit (Accurate Biotechnology, Hunan, China) according to the manufacturer’s instructions. The extracted DNA samples were submitted to TianGen Biotech Co., Ltd. (Beijing, China) for WGS (Supplementary Data Sheet 1). The sequencing process was as follows: qualified DNA samples were randomly sheared into ~350 bp fragments using a Covaris ultrasonicator. The DNA fragments were then subjected to end-repair, A-tailing, adapter ligation, purification, and PCR amplification to construct sequencing libraries. Library concentration was preliminarily quantified with a Qubit 2.0 fluorometer and diluted to 2 ng/μL. The insert size was assessed using an Agilent 2,100 Bioanalyzer. Quantitative PCR (qPCR) was performed to accurately determine the effective library concentration to ensure sequencing quality. Sequencing was performed on the Illumina HiSeq PE150 platform, using a paired-end 150 bp strategy with a 350 bp short-insert library and a bacterial resequencing coverage depth of ≥10×. Raw reads with low-quality scores (Q score ≥20) were filtered to obtain high-quality clean reads. *De novo* assembly of paired-end reads was carried out using CLC Genomics Workbench v21.0.1. The assembled genomes were then annotated using Prokka (v1.14.x).

### 2.3 ARG detection and classification based on WGS data

ARGs were identified using the ResFinder (v4.1) database. A minimum identity threshold of 90% and minimum coverage of 60% were applied for gene detection. Resistance determinants were categorized according to their antibiotic classes, including *β*-lactams (e.g., *bla*KPC, *bla*CTX-M, *bla*TEM, and *bla*SHV), aminoglycosides (e.g., aac(3), aac(6′), *aph*, *aadA*, and *rmtB*), quinolones (*qnr* genes and *oqxAB*), sulfonamides (*sul1*, *sul2*), tetracyclines (tetA, *tetB*), macrolides (*mph*, *msr*), phenicols (*cat*, *floR*), and fosfomycin (*fosA*), among others.

### 2.4 ST typing

The sequence types (STs) of all 52 genomes were determined using a custom-developed sequence typing tool, **ST_tool**. This analysis utilized six housekeeping genes from *Serratia* species, obtained from the pubMLST database using the PubMLST scheme for *S. marcescens*, and analyzed via the multilocus sequence typing tool (MLST) (Torsten Seemann, https://github.com/tseemann/mlst) (accessed on September 15, 2024). The genomes were aligned to these housekeeping gene sequences using BLASTn, with an identity threshold of 100% and a coverage threshold of 100%. The resulting BLAST matches were then compared to the profile files from pubMLST to assign the corresponding ST. The ST results were recorded in an Excel file.

### 2.5 Virulence gene analysis

Virulence gene profiles were determined by comparing assembled genomes to the Virulence Factor Database (VFDB) and BIGSdb-Kp using ABRicate (v1.x) with a minimum sequence identity threshold of 90% and coverage threshold of 80%. The presence of key virulence determinants were recorded. To explore the distribution patterns of virulence genes among the isolates, a binary presence/absence matrix was constructed, and hierarchical clustering was performed in R (v4.x) using the Jaccard similarity index. Co-occurrence of virulence factors was analyzed with Fisher’s exact test, and their relationship with sequence types (STs) and resistance profiles was evaluated using chi-square tests. Phylogenetic relationships were inferred using core-genome SNPs identified by Snippy (v4.x), and virulence gene content was visualized alongside the phylogeny with iTOL.

### 2.6 Phylogenetic tree construction

The phylogenetic tree for the 52 *Serratia marcescens* isolates was constructed using Gubbins and IQ-TREE software. First, a multi-sequence alignment was performed using Snippy software with the full genome of *Serratia marcescens* subsp. *marcescens* ATCC 13880 (GCA_017654245.1) as the reference genome. This alignment was then processed with Gubbins to infer the core genome alignment and exclude recombination events. The phylogenetic tree was built using IQ-TREE with the GTR model and 1000 bootstrap replicates for branch reliability (Seemann, 2014).

### 2.7 Bacterial growth assay

To evaluate potential fitness costs and growth variability among outbreak clones, bacterial growth assays were conducted. A single freshly grown bacterial colony was inoculated into 5 mL of LB broth and incubated overnight at 150 rpm for 16–18 h. Then, 20 μL of the overnight culture was added to 2 mL of fresh LB broth, mixed well, and 100 μL of this suspension was transferred to a 96-well microplate with four replicates per sample. Optical density (OD620) was measured hourly using a multifunctional microplate reader, and the growth data were recorded. Bacterial growth curves were plotted using GraphPad Prism 5 to evaluate the *in vitro* growth capacity (Hua et al., 2021).

### 2.8 Biofilm formation assay

Because biofilm formation is a recognized mechanism of persistence in hospital environments, the ability of CRSM isolates to form biofilms was assessed. A single freshly grown bacterial colony was inoculated into 5 mL of LB broth and incubated overnight with shaking. Then, 20 μL of the overnight culture was added to 2 mL of fresh LB broth, mixed well, and 100 μL of the suspension was transferred to a 96-well microplate with four replicates per sample. After incubation at 37 °C for 4–24 h, the liquid was discarded, and the wells were gently washed by immersing the plate in sterile water, swirling gently, and discarding the wash; this was repeated twice. Each well was then stained with 125 μL of 0.1% crystal violet solution and incubated at room temperature for 10–15 min. The plate was then immersed in water 3–4 times, and excess stain was vigorously shaken out onto absorbent paper. After air-drying for 2 h, 125 μL of 30% glacial acetic acid was added to each well and incubated at room temperature for 15 min to dissolve the stain. Then, 125 μL of the solution was transferred to a new 96-well plate, and the absorbance was measured at OD550 nm, with 30% glacial acetic acid serving as the blank control (Ernst et al., 2020). Results were analyzed and plotted using GraphPad Prism 5.

### 2.9 *In vitro* serum bactericidal assay

Since serum resistance reflects the ability of pathogens to evade innate immunity, an *in vitro* bactericidal assay was performed. Exponentially growing bacterial cultures were adjusted to a turbidity equivalent to 0.5 McFarland standard using sterile saline and further diluted to 0.5 × 10^6^ CFU/mL. The bacterial suspension was mixed with non-immune human serum at a ratio of 1:3 (t = 0) and incubated at 37 °C for 3 h. At 0, 1, 2, and 3 h, 100 μL samples were collected, serially diluted in saline, and plated to determine viable counts by colony counting. Each experiment was performed in triplicate (Han et al., 2022). The survival rate of bacteria at different time points was calculated to assess serum bactericidal resistance. Data were analyzed and visualized using GraphPad Prism.

### 2.10 Statistical analysis

All statistical analyses were performed using GraphPad Prism version 5.0 (GraphPad Software, United States) and R version 4.x. For growth curves, biofilm formation, and serum resistance assays, data were obtained from at least three independent experiments with technical replicates. Differences between two groups were evaluated using Student’s *t*-test, while comparisons among multiple groups or clones were assessed by one-way analysis of variance (ANOVA) followed by Tukey’s *post hoc* test. For categorical data, including the distribution and co-occurrence of virulence genes, Fisher’s exact test or chi-square test was applied as appropriate. *p*-values < 0.05 were considered statistically significant.

## 3 Result

### 3.1 Diverse and multifactorial antibiotic resistance gene landscape in CRSM outbreak strains

WGS-based bacterial identification was consistent with the MALDI-TOF results for all isolates, confirming the accuracy of the genomic identification approach. All 52 outbreak isolates carried *bla*KPC-2 (52/52), confirming its central role in carbapenem resistance. Notably, a subset of isolates also carried additional carbapenemase genes, including *bla*NDM-1 (*n* = 3), thereby reinforcing the presence of double-carbapenemase-producing isolates within the outbreak. Beyond carbapenemases, the majority of isolates harbored *bla*SRT-1 (48/52), *bla*CTX-M-14 (42/52), *bla*LAP-2 (37/52), and *bla*TEM-1B (25/52). while multiple *bla*SHV variants (11/52) were detected at lower frequencies. Multiple aminoglycoside resistance genes, were found, with *rmtB*, and *armA* in lower frequencies. Quinolone resistance was widespread, with *qnrS1* present in all isolates, and additional determinants such as *qnrB* variants and *oqxAB* detected in subsets. Aminoglycoside resistance genes were also prevalent, though *rmtB* and *armA* occurred sporadically. Collectively, these findings highlight a multifactorial resistance gene repertoire across outbreak isolates.

### 3.2 Virulence gene profiles and clonal associations in CRSM outbreak isolates revealed by WGS

**Figure 1:**
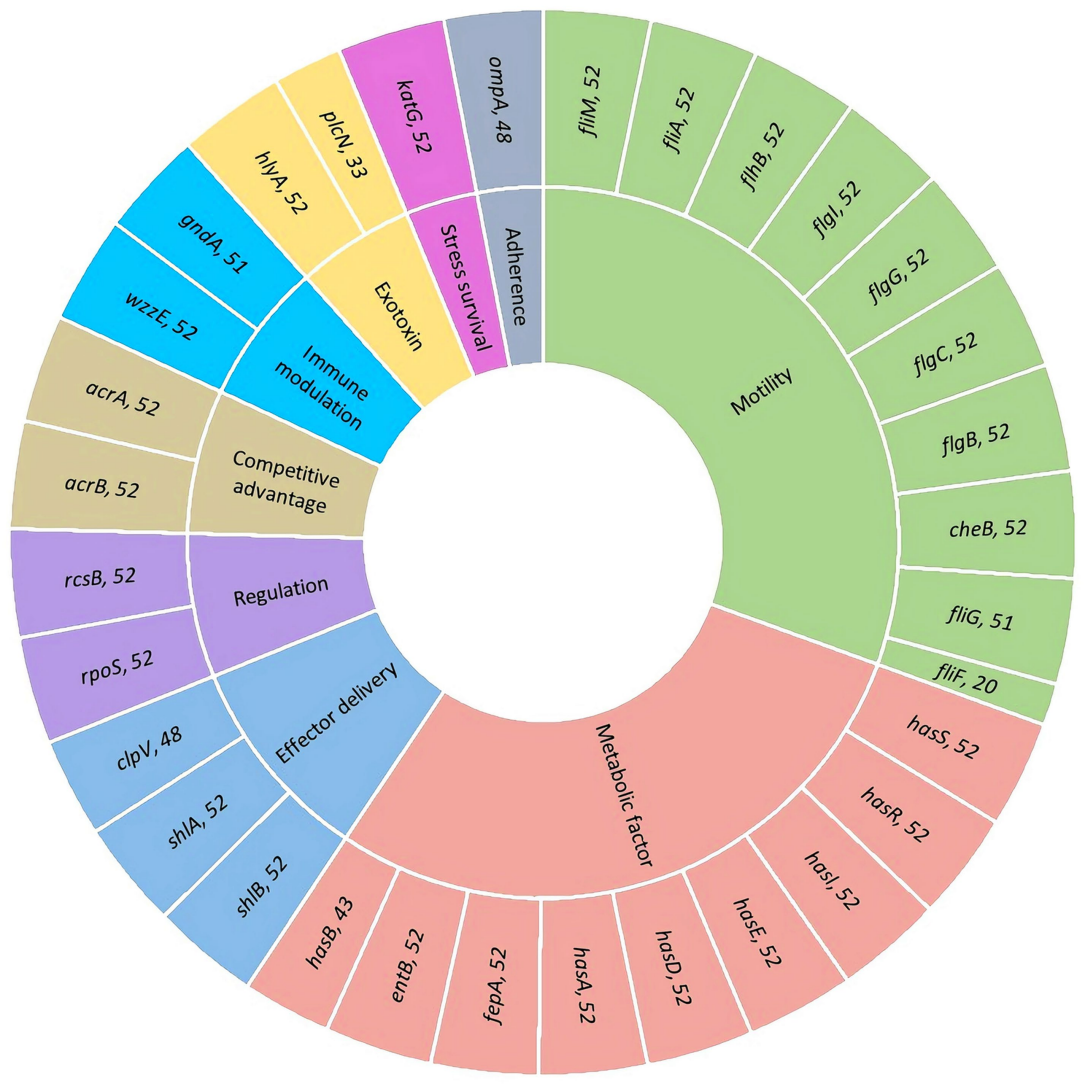
Functional classification and distribution of virulence genes identified in 52 carbapenem-resistant *Serratia marcescens* isolates. The sunburst diagram illustrates the categorization of virulence-associated genes based on their functional roles, including motility (green), metabolic factors (red), effector delivery (blue), regulation (purple), competitive advantage (tan), immune modulation (light blue), exotoxins (yellow), stress survival (gray), and adherence (dark gray). The outer ring displays individual gene names along with the number of CRSM isolates in which each gene was detected.

All isolates carried a broad array of virulence-associated genes spanning adhesion, motility, toxin secretion, iron acquisition, and immune modulation. Of note, the toxin-associated gene *plcN* was enriched in ST595 (24/25), while the motility-related gene *fliF* was predominant in ST640 (17/17), suggesting clonal enrichment of lineage-specific virulence traits. Universal presence of regulators such as *katG*, *rcsB*, and *rpoS* indicated strong stress-adaptation potential (Figure 1). These data imply that distinct virulence signatures may synergize with resistance to support persistence of dominant clones.

### 3.3 Dominance and phylogenetic structure of ST595 and ST640 among CRSM outbreak isolates

**Figure 2:**
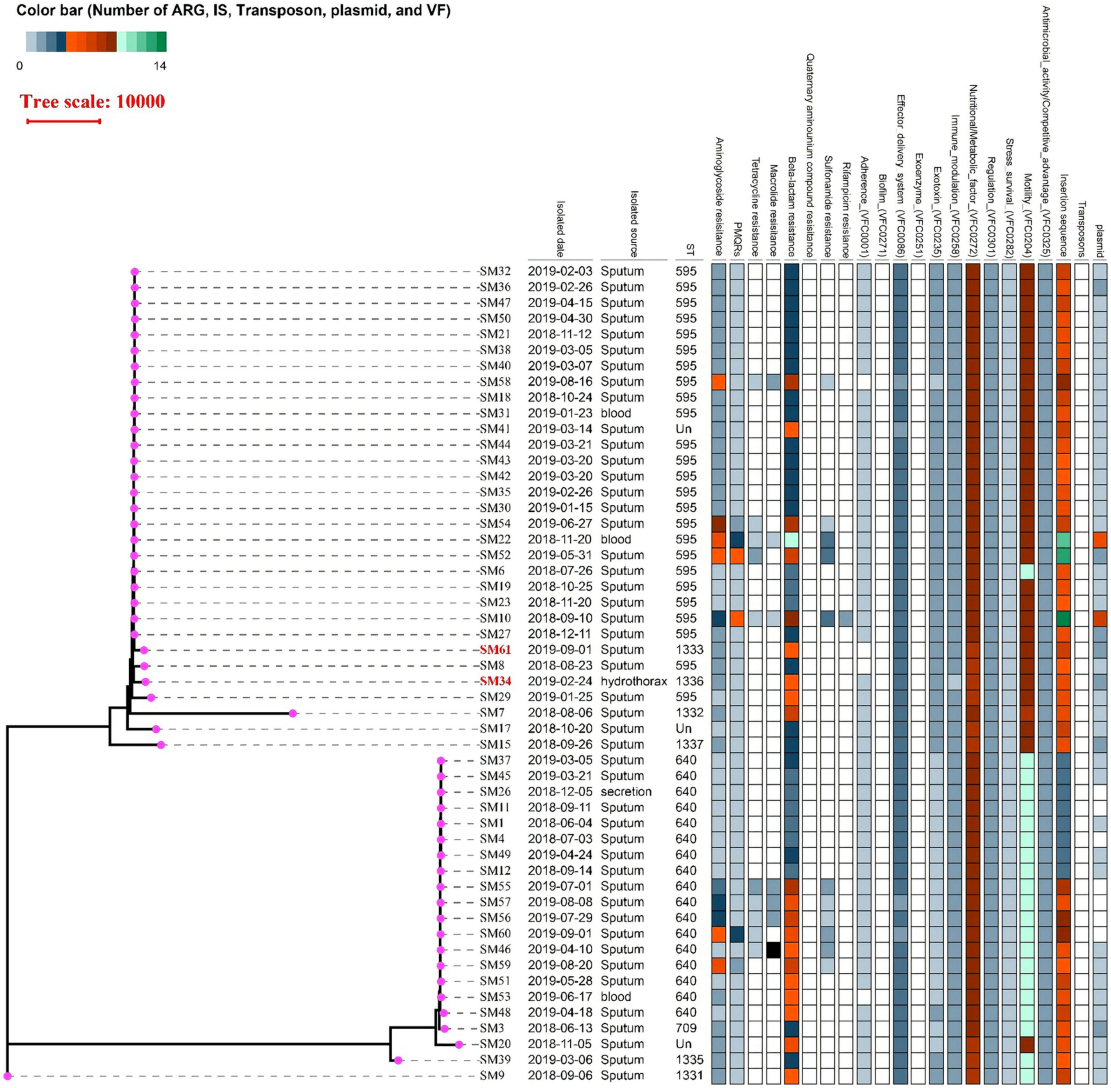
Phylogenetic tree and genomic feature heatmap of 52 *Serratia marcescens* isolates from a hospital outbreak. The tree was constructed using Gubbins and IQ-TREE, based on core genome single nucleotide polymorphisms (SNPs). The color bar on the left represents the number of antibiotic resistance genes (ARGs), insertion sequences (IS), transposons, plasmids, and virulence factors (VFs) identified in each isolate. The heatmap on the right shows the presence (colored blocks) or absence (gray) of these genomic features across all isolates. Isolates are labeled with their respective sequence types (STs), with ST595 and ST640 forming the major clonal groups.

MLST identified two major epidemic clones: ST595 (25/52) and ST640 (17/52). Three isolates were unclassified, which could be due to factors such as genetic variability, the limitations of the current MLST database not encompassing all strains, or mutations in the housekeeping genes required for ST typing. and the remaining isolates belonged to diverse minor STs. This distribution indicates that ST595 and ST640 accounted for the majority of the outbreak population, suggesting their enhanced epidemic potential. Core genome phylogeny revealed two distinct evolutionary lineages corresponding to ST595 and ST640. ST595 isolates were closely related, supporting the idea of clonal expansion during the outbreak. In contrast, ST640 isolates displayed greater diversity in their ARG profiles, suggesting multiple introductions or diversification events. However, it is noteworthy that two isolates had discrepancies between their STs and phylogenetic tree positions, which could be due to factors such as horizontal gene transfer or recombination events (Zhang et al., 2024). Several minor STs (e.g., ST1331, ST1335, ST1337) formed peripheral branches, reflecting genetic heterogeneity and possible independent lineages (Figure 2). The frequent detection of mobile genetic elements indicates a role for horizontal gene transfer in shaping the resistance and virulence profiles of these clones, contributing to the dissemination and spread of these lineages during the outbreak.

### 3.4 Phenotypic heterogeneity in growth, biofilm formation, and serum resistance

Phenotypic assays revealed notable heterogeneity among isolates. Some strains displayed rapid growth and robust biofilm formation, while others exhibited attenuated proliferation and weak biofilm capacity. Importantly, a subset of isolates demonstrated enhanced serum resistance, maintaining high survival despite complement exposure. These traits were not uniformly distributed but tended to align with the epidemic clones, suggesting that high-risk phenotypes—such as strong biofilm production and immune evasion—may have facilitated persistence and spread during the outbreak.

#### 3.4.1 Variation in growth rates of ST595 and ST640 strains

**Figure 3:**
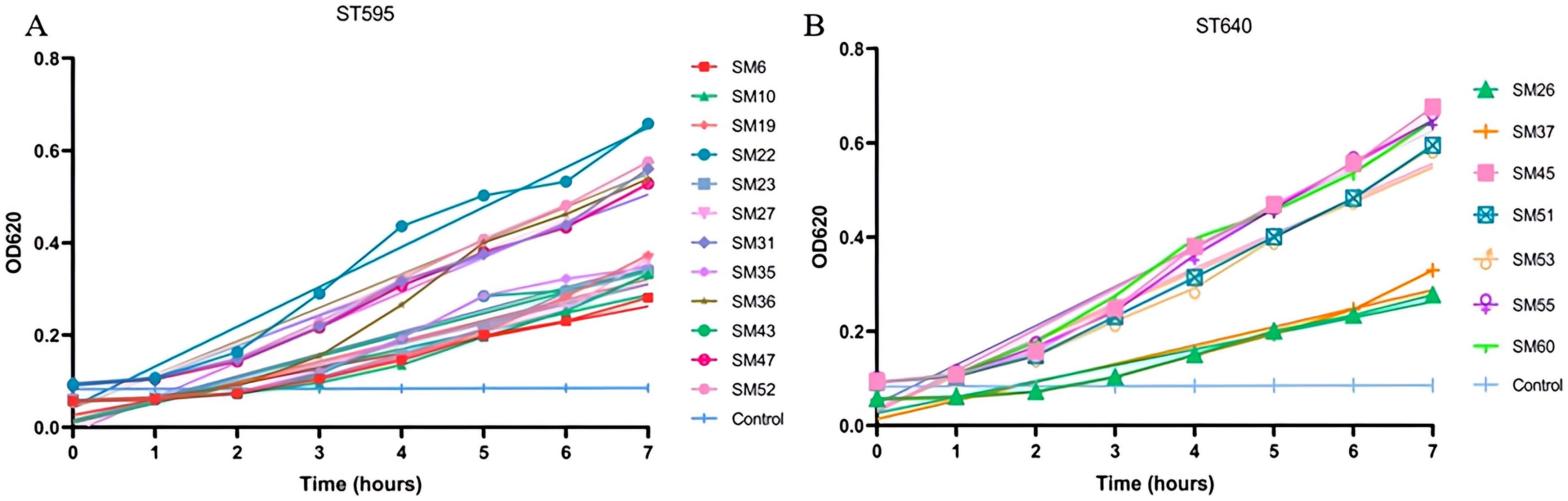
Bacterial growth kinetics of *Serratia marcescens* isolates from different sequence types (ST595 and ST640). **(A)** Shows the growth of ST595 isolates, and **(B)** shows the growth of ST640 isolates. The y-axis represents the optical density at 620 nm (OD620), indicating bacterial growth, and the x-axis represents time (in hours) over a 7-h period. Different isolates are indicated by various symbols and colors, with the control group included for comparison.

The bacterial growth assay results show significant variability in the growth rates of the ST595 and ST640 strains. In the ST595 group (Panel A), strains like SM22, SM52, and SM31 exhibit the highest growth rates, while strains such as SM6, SM10, and SM23 show slower growth. In the ST640 group (Panel B), strains like SM45, SM55, and SM60 display the fastest growth, while SM26 grows at a much slower rate (Figure 3). Overall, these results indicate differences in growth characteristics across the strains, with some showing enhanced proliferation.

#### 3.4.2 Variation in biofilm production among ST595 and ST640 strains

**Figure 4:**
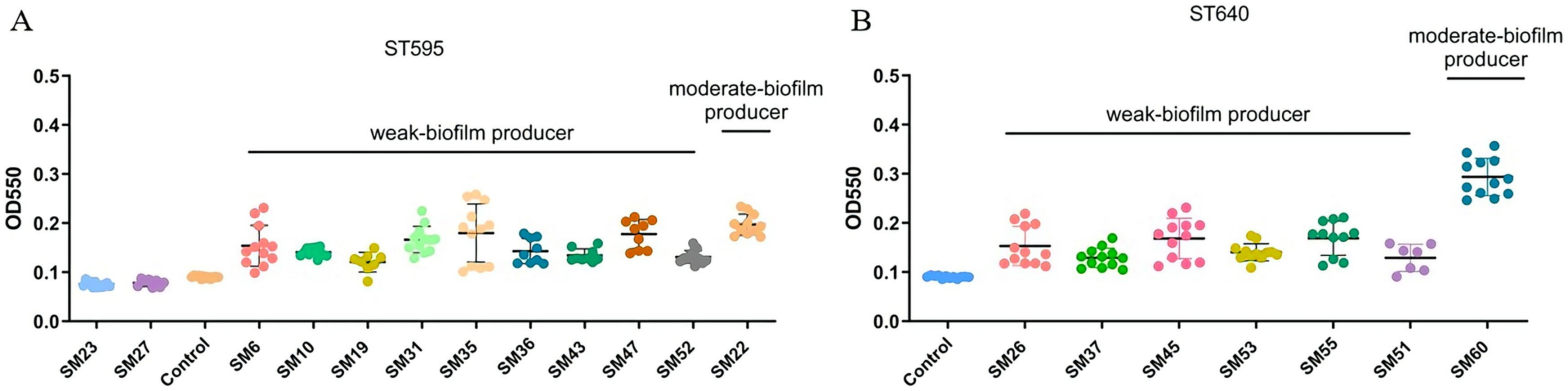
Biofilm formation of *Serratia marcescens* isolates from different sequence types (ST595 and ST640). **(A)** Shows the results for ST595 isolates, and **(B)** shows the results for ST640 isolates. The y-axis represents the optical density at 550 nm (OD550), a measure of biofilm formation, and the x-axis represents the different isolates tested, including the control. Isolates are grouped into weak-biofilm producers and moderate-biofilm producers based on their OD550 values.

The biofilm formation analysis shows significant variability in biofilm production across both ST595 and ST640 strains. In the ST595 group (Panel A), strains such as SM6, SM10, SM19, SM31, SM35, SM36, SM43, SM47 and SM52 are classified as weak biofilm producers, while SM22 demonstrate moderate biofilm production. Similarly, in the ST640 group (Panel B), strains like SM26, SM37, SM45, SM53, SM55 and SM51 are weak biofilm producers, whereas SM60 stand out as moderate biofilm producers (Figure 4). Overall, these results highlight the diverse biofilm-forming abilities of the strains, which may influence their pathogenicity and persistence. Further investigation into the mechanisms driving these differences is needed.

#### 3.4.3 Variability in antibacterial serum response among ST595 and ST640 strains

**Figure 5:**
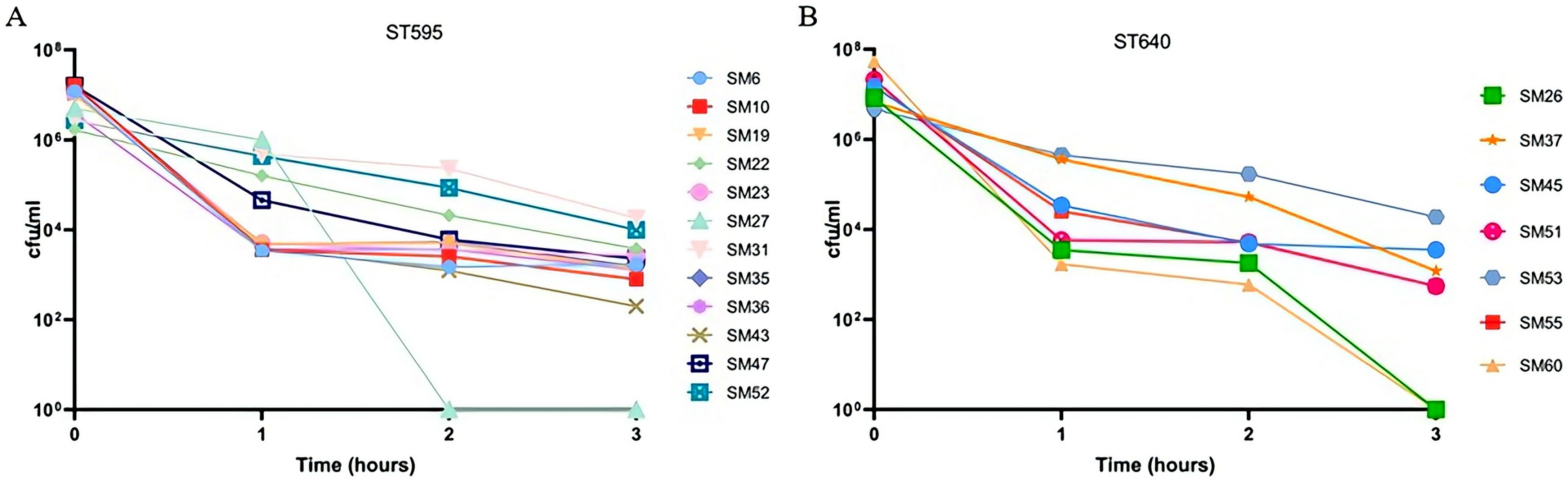
Antibacterial serum killing assay of *Serratia marcescens* isolates from different sequence types (ST595 and ST640). **(A)** Shows the results for ST595 isolates, and **(B)** shows the results for ST640 isolates. The y-axis represents the colony-forming units per milliliter (CFU/ml) over a 3-h period, and the x-axis represents time (in hours) post-incubation with serum. Different isolates are denoted by various symbols and colors.

The results of the antibacterial serum killing assay show a clear reduction in CFU/ml for both ST595 and ST640 strains over time. For ST595 (Panel A), strains such as SM31 and SM52 demonstrate slower declines, suggesting a higher resistance to the serum, while strains like SM27, SM43, and SM10 show more significant reductions, indicating higher susceptibility. In ST640 (Panel B), most strains, including SM26 and SM60, exhibit a rapid decline in CFU/ml, but strains like SM53, SM45, SM37, SM55, and SM51 show relatively higher resistance, maintaining a higher bacterial count over the 3-h period (Figure 5). Overall, the serum demonstrates broad antibacterial activity, but variability in susceptibility exists, and further research may be needed to investigate the factors contributing to resistance in certain strains.

## 4 Discussion

The present study provides an integrated genomic and phenotypic analysis of 52 CRSM clinical isolates obtained during a documented nosocomial outbreak, thereby offering a rare opportunity to investigate the interplay between resistance, virulence, and clonal dissemination in a real-world hospital setting. By combining WGS with targeted phenotypic assays, we identified two dominant epidemic clones, ST595 and ST640, and delineated their distinct resistance and virulence features.

Our genomic analysis revealed a multifactorial resistome across outbreak strains, with universal carriage of *bla*KPC-2 and frequent detection of ESBLs and plasmid-borne aminoglycoside and quinolone resistance genes. Such redundancy provides both therapeutic challenges and selective advantages under antimicrobial pressure, favoring clonal persistence.

Globally, CRSM exhibits marked clonal and mechanistic heterogeneity, with multiple STs independently acquiring carbapenemases and spreading in hospital settings. Previous reports have highlighted the prevalence of KPC- and NDM-producing *S. marcescens* in South America and Europe, the dissemination of GIM-1 producers in Germany, and the detection of SME enzymes in Canada and the UK. Although ST595 and ST640 have not been widely reported as dominant international lineages, our findings expand the known diversity of CRSM epidemic clones. These clones add new insights to the global scenario of CRSM transmission and resistance, linking our local outbreak to broader international trends. Their resistomes—dominated by KPC-2, with additional *β*-lactamase, aminoglycoside, and quinolone determinants—mirror global resistance trends, while their virulence gene repertoires overlap with canonical *S. marcescens* traits such as hemolysins, secretion systems, and iron acquisition loci. These comparisons reinforce that CRSM persistence and spread are driven by combinations of resistance and virulence factors, rather than a single lineage-specific mechanism.

The predominance of ST595 and ST640, together accounting for nearly 81% of isolates, suggests clonal expansion as the driving force of the outbreak. ST595 showed close phylogenetic clustering and frequent isolation from respiratory specimens, consistent with a regionally circulating epidemic lineage selected by strong antibiotic or ecological pressures. In contrast, ST640 exhibited greater accessory gene diversity and phylogenetic spread, suggesting repeated introductions from environmental or zoonotic reservoirs. The coexistence of rare sequence types further highlights ongoing diversification and the potential for inter-facility or interspecies transmission.

All CRSM strains shared a conserved set of core virulence genes, including *acrA/B*, *shlA/B*, *hlyA*, *cheB*, *flg* family members, and iron acquisition systems (*has*, *entB*, *fepA*), highlighting a baseline pathogenic potential across the outbreak population. These genes encode factors critical for adherence, immune modulation, toxin delivery, and iron scavenging—functions essential for *S. marcescens* colonization and survival in host tissues during nosocomial spread (Bolourchi et al., 2022; González et al., 2020; Prado et al., 2022). The universal presence of stress response regulators *katG*, *rcsB*, and *rpoS* further underscores their adaptive plasticity, enabling persistence under antibiotic pressure and in the hostile hospital environment (Ballaben et al., 2025). Interestingly, specific virulence traits displayed clonal enrichment during the outbreak. For instance, the *plcN* gene, a phospholipase toxin gene (Truşcă et al., 2023), was predominantly associated with ST595, suggesting vertical inheritance or recent acquisition within this epidemic lineage. This points to the possibility that certain virulence traits are retained or enhanced through clonal expansion, reflecting selective pressures within the hospital environment. In contrast, the flagellar gene *fliF* was concentrated in ST640 strains, potentially enhancing motility and colonization of different hospital niches. This suggests that virulence evolution in *S. marcescens* is shaped by both clonal background and ecological pressures specific to the hospital setting, where motility and colonization ability are important for persistence and transmission. These findings demonstrate that the evolution of virulence factors is not random but instead is influenced by both genetic background and environmental factors, including hospital-specific conditions such as antimicrobial pressure and host immune responses.

Phenotypic assays provided additional insights into outbreak persistence (Liu et al., 2023; Cruz et al., 2024; Ferguson et al., 2023). Targeted phenotypic assays were designed to further assess traits relevant to outbreak persistence. Biofilm formation—a key factor in environmental persistence and device-associated infections—was moderate to strong in a subset of isolates (Lin et al., 2016; Mayhew et al., 2024), including SM22 and SM60. The co-occurrence of robust biofilm production and serum resistance in certain isolates, suggests the emergence of “dual-threat” phenotypes optimized for both environmental survival and immune evasion, traits that can amplify the impact of clonal dissemination in a hospital outbreak context.

Interestingly, the phenotypic profiles of growth rate, biofilm formation, and serum resistance did not fully align with the phylogenetic relationships shown in Figure 2. This discrepancy may reflect genetic variation not captured in the phylogenetic analysis (e.g., small-scale mutations or variations in mobile genetic elements), which could modulate these traits without altering the overall phylogeny. Additionally, gene regulation and environmental adaptation may further contribute to the observed phenotypic heterogeneity. These findings underscore the complexity of linking genotype to phenotype in *S. marcescens* and highlight that phenotypic diversity may evolve through multiple pathways beyond clonal lineage.

Variability in growth dynamics reflected potential fitness differences, while enhanced biofilm formation and serum resistance in subsets of isolates identified “high-risk” phenotypes that combine environmental persistence with immune evasion. The clustering of these traits within ST595 and ST640 suggests that the success of these clones was driven not only by resistance but also by synergistic phenotypic advantages.

Serum killing assays further stratified the immune evasion capacity of the outbreak strains. While most isolates were efficiently cleared by complement-mediated lysis, a subset—including SM53, SM52, and SM31—maintained viable populations after prolonged serum exposure. The clustering of serum-resistant isolates within the dominant outbreak STs implies that this trait may have been selectively enriched during transmission (Ding et al., 2025), enhancing bloodstream infection potential and complicating outbreak containment.

The convergence of multidrug resistance, enriched virulence gene profiles, biofilm-forming ability, and serum resistance within epidemic clones ST595 and ST640 underscores their classification as high-risk lineages. The outbreak setting amplifies the clinical significance of these findings: the combination of resistance and virulence likely facilitated both persistence in the hospital environment and patient-to-patient transmission. The abundance of mobile genetic elements among these isolates further raises concern for horizontal gene transfer during outbreaks, potentially accelerating the evolution of future epidemic clones.

This study has several limitations. The outbreak was confined to a single center, which may limit the generalizability of the findings. Lack of detailed clinical metadata precluded correlation between bacterial traits and patient outcomes. *In vitro* assays may not fully capture *in vivo* dynamics, and the presence of virulence genes does not confirm their expression or contribution to pathogenicity. Although resistant determinants were detected using WGS, additional molecular confirmation of these genes was not conducted. In addition, we were unable to reliably distinguish intrinsic from acquired resistance genes in *S. marcescens*, as current databases are not optimized for this species. Inclusion of carbapenem-susceptible reference isolates, long-read sequencing, and curated genomes will be needed in future studies to address this gap. Overall, this work is descriptive in nature, providing a baseline characterization of CRSM isolates that lays the groundwork for hypothesis-driven studies to clarify mechanisms of resistance and virulence. Nonetheless, by capturing the genomic and phenotypic signatures of CRSM during a verified hospital outbreak, this study provides critical insight into the mechanisms that enable clonal expansion and persistence in healthcare settings. These findings can inform hospital surveillance strategies by identifying high-risk lineages and resistance determinants that warrant targeted screening, guide outbreak preparedness by highlighting the genetic traits linked to persistence and dissemination, and support therapeutic decision-making by clarifying the resistance landscape relevant to antimicrobial stewardship. Future work should include multicenter surveillance, incorporation of molecular carbapenemase confirmation, and *in vivo* validation to further elucidate the epidemiological and pathogenic roles of these high-risk CRSM clones.

## 5 Conclusion

In summary, this study provides an integrated genomic and phenotypic characterization of 52 CRSM isolates collected during a documented nosocomial outbreak, identifying ST595 and ST640 as the dominant epidemic clones. These lineages exhibited multifactorial resistomes with universal carriage of *bla*KPC-2 and additional resistance genes, along with lineage-specific virulence traits such as *plcN* in ST595 and *fliF* in ST640. Phenotypic assays revealed considerable heterogeneity in growth dynamics, biofilm formation, and serum resistance, while certain isolates combining high-risk traits like robust biofilm production and immune evasion, which likely contributed to persistence and clonal dissemination in the hospital environment. Our findings emphasize the importance of real-time genomic surveillance and functional profiling in outbreak settings, highlighting the need for enhanced screening, cohorting strategies, and targeted antimicrobial interventions to control the spread of high-risk CRSM clones. These results provide actionable insights for infection control and antimicrobial stewardship strategies in healthcare facilities, aimed at optimizing therapy and preventing further dissemination.

## Data Availability

The datasets presented in this study can be found in online repositories. The names of the repository/repositories and accession number(s) can be found in the article/Supplementary material.
