# Supplementary material for: Emergence of high-risk ST595 and ST640 clones of carbapenem-resistant Serratia marcescens: insights from genomic and virulence profiling during a nosocomial epidemic
Source: Front Microbiol. 2025 Oct 9;16:1681911. doi: 10.3389/fmicb.2025.1681911 (PMC12546152; doi:10.3389/fmicb.2025.1681911)
Supplement: Supplementary file 1 [file Table_1.docx]

**Supplementary Table 1.** The minimum inhibitory concentrations (mg/L) of 52 *Serratia marcescens* strains

| Strain | ST | TZP | CAZ | FEP | ATM | IPM | AK | CN10 | CIP | LEV | SXT |
| --- | --- | --- | --- | --- | --- | --- | --- | --- | --- | --- | --- |
| SM10 | 595 | 128 (R) | 32 (R) | 16 (R) | 64 (R) | 16 (R) | 4 (S) | 2 (S) | 4 (R) | 8 (R) | 20 (S) |
| SM18 | 595 | 128 (R) | 8 (I) | 64 (R) | 64 (R) | 16 (R) | 2 (S) | 1 (S) | 4 (R) | 8 (R) | 20 (S) |
| SM19 | 595 | 128 (R) | 32 (R) | 64 (R) | 64 (R) | 16 (R) | 4 (S) | 4 (I) | 4 (R) | 8 (R) | 20 (S) |
| SM21 | 595 | 128 (R) | 8 (I) | 64 (R) | 64 (R) | 16 (R) | 8 (I) | 16 (R) | 4 (R) | 4 (R) | 20 (S) |
| SM22 | 595 | 128 (R) | 64 (R) | 32 (R) | 64 (R) | 4 (R) | 8 (I) | 4 (I) | 0.5 (S) | 0.5 (S) | 320 (R) |
| SM23 | 595 | 128 (R) | 8 (I) | 64 (R) | 64 (R) | 16 (R) | 4 (S) | 1 (S) | 4 (R) | 8 (R) | 20 (S) |
| SM27 | 595 | 128 (R) | 1 (S) | 64 (R) | 64 (R) | 16 (R) | 4 (S) | 16 (R) | 4 (R) | 4 (R) | 20 (S) |
| SM29 | 595 | 128 (R) | 0.5 (S) | 64 (R) | 64 (R) | 16 (R) | 8 (I) | 1 (S) | 4 (R) | 8 (R) | 20 (S) |
| SM30 | 595 | 128 (R) | 0.5 (S) | 128 (R) | 64 (R) | 16 (R) | 2 (S) | 16 (R) | 4 (R) | 4 (R) | 20 (S) |
| SM31 | 595 | 128 (R) | 8 (I) | 64 (R) | 64 (R) | 16 (R) | 2 (S) | 2 (S) | 1 (S) | 1 (I) | 20 (S) |
| SM32 | 595 | 128 (R) | 8 (I) | 64 (R) | 64 (R) | 16 (R) | 4 (S) | 16 (R) | 2 (R) | 2 (R) | 20 (S) |
| SM35 | 595 | 64 (R) | 0.5 (S) | 8 (SDD) | 64 (R) | 16 (R) | 4 (S) | 16 (R) | 4 (R) | 2 (R) | 20 (S) |
| SM36 | 595 | 64 (R) | 16 (R) | 8 (SDD) | 64 (R) | 16 (R) | 4 (S) | 16 (R) | 4 (R) | 2 (R) | 20 (S) |
| SM38 | 595 | 64 (R) | 8 (I) | 8 (SDD) | 64 (R) | 16 (R) | 4 (S) | 16 (R) | 4 (R) | 4 (R) | 20 (S) |
| SM40 | 595 | 64 (R) | 8 (I) | 8 (SDD) | 64 (R) | 16 (R) | 4 (S) | 16 (R) | 4 (R) | 2 (R) | 20 (S) |
| SM42 | 595 | 64 (R) | 8 (I) | 8 (SDD) | 64 (R) | 16 (R) | 2 (S) | 16 (R) | 2 (R) | 2 (R) | 20 (S) |
| SM43 | 595 | 64 (R) | 8 (I) | 8 (SDD) | 64 (R) | 16 (R) | 4 (S) | 16 (R) | 2 (R) | 2 (R) | 20 (S) |
| SM44 | 595 | 128 (R) | 8 (I) | 8 (SDD) | 64 (R) | 16 (R) | 4 (S) | 16 (R) | 4 (R) | 4 (R) | 20 (S) |
| SM47 | 595 | 64 (R) | 8 (I) | 8 (SDD) | 64 (R) | 16 (R) | 4 (S) | 16 (R) | 4 (R) | 4 (R) | 20 (S) |
| SM50 | 595 | 128 (R) | 8 (I) | 32 (R) | 64 (R) | 16 (R) | 4 (S) | 16 (R) | 4 (R) | 4 (R) | 20 (S) |
| SM52 | 595 | 64 (R) | 8 (I) | 16 (R) | 64 (R) | 16 (R) | 4 (S) | 16 (R) | 4 (R) | 2 (R) | 20 (S) |
| SM54 | 595 | 64 (R) | 8 (I) | 64 (R) | 64 (R) | 16 (R) | 4 (S) | 16 (R) | 4 (R) | 4 (R) | 20 (S) |
| SM58 | 595 | 64 (R) | 0.5 (S) | 64 (R) | 16 (R) | 4 (R) | 16 (R) | 4 (I) | 1 (S) | 1 (I) | 20 (S) |
| SM6 | 595 | 128 (R) | 64 (R) | 64 (R) | 64 (R) | 16 (R) | 8 (I) | 1 (S) | 4 (R) | 8 (R) | 20 (S) |
| SM8 | 595 | 128 (R) | 0.25 (S) | 16 (R) | 64 (R) | 16 (R) | 8 (I) | 1 (S) | 4 (R) | 8 (R) | 20 (S) |
| SM1 | 640 | 64 (R) | 0.5 (S) | 16 (R) | 64 (R) | 16 (R) | 2 (S) | 1 (S) | 4 (R) | 8 (R) | 20 (S) |
| SM11 | 640 | 128 (R) | 0.5 (S) | 16 (R) | 32 (R) | 16 (R) | 16 (R) | 16 (R) | 4 (R) | 4 (R) | 20 (S) |
| SM12 | 640 | 128 (R) | 0.25 (S) | 16 (R) | 64 (R) | 16 (R) | 16 (R) | 1 (S) | 4 (R) | 8 (R) | 20 (S) |
| SM26 | 640 | 128 (R) | 8 (I) | 64 (R) | 64 (R) | 16 (R) | 4 (S) | 1 (S) | 4 (R) | 8 (R) | 20 (S) |
| SM37 | 640 | 128 (R) | 0.25 (S) | 4 (SDD) | 64 (R) | 16 (R) | 16 (R) | 2 (S) | 4 (R) | 8 (R) | 20 (S) |
| SM4 | 640 | 64 (R) | 0.25 (S) | 2 (S) | 64 (R) | 16 (R) | 2 (S) | 1 (S) | 4 (R) | 4 (R) | 20 (S) |
| SM45 | 640 | 128 (R) | 2 (S) | 4 (SDD) | 64 (R) | 16 (R) | 2 (S) | 1 (S) | 4 (R) | 4 (R) | 20 (S) |
| SM46 | 640 | 64 (R) | 1 (S) | 4 (SDD) | 64 (R) | 16 (R) | 2 (S) | 1 (S) | 4 (R) | 4 (R) | 20 (S) |
| SM48 | 640 | 64 (R) | 8 (I) | 16 (R) | 64 (R) | 16 (R) | 4 (S) | 16 (R) | 4 (R) | 2 (R) | 20 (S) |
| SM49 | 640 | 64 (R) | 8 (I) | 4 (SDD) | 64 (R) | 16 (R) | 2 (S) | 1 (S) | 4 (R) | 4 (R) | 20 (S) |
| SM51 | 640 | 64 (R) | 8 (I) | 8 (SDD) | 64 (R) | 16 (R) | 4 (S) | 16 (R) | 4 (R) | 2 (R) | 20 (S) |
| SM53 | 640 | 64 (R) | 8 (I) | 8 (SDD) | 64 (R) | 16 (R) | 4 (S) | 16 (R) | 4 (R) | 2 (R) | 20 (S) |
| SM55 | 640 | 64 (R) | 0.25 (S) | 64 (R) | 64 (R) | 16 (R) | 2 (S) | 1 (S) | 4 (R) | 4 (R) | 320 (R) |
| SM56 | 640 | 64 (R) | 0.25 (S) | 64 (R) | 64 (R) | 16 (R) | 2 (S) | 1 (S) | 4 (R) | 4 (R) | 20 (S) |
| SM57 | 640 | 64 (R) | 1 (S) | 4 (SDD) | 64 (R) | 16 (R) | 2 (S) | 16 (R) | 4 (R) | 4 (R) | 20 (S) |
| SM59 | 640 | 64 (R) | 0.5 (S) | 4 (SDD) | 64 (R) | 16 (R) | 2 (S) | 1 (S) | 4 (R) | 4 (R) | 20 (S) |
| SM60 | 640 | 64 (R) | 64 (R) | 8 (SDD) | 64 (R) | 16 (R) | 4 (S) | 2 (S) | 4 (R) | 8 (R) | 20 (S) |
| SM3 | 709 | 64 (R) | 0.5 (S) | 2 (S) | 64 (R) | 16 (R) | 4 (S) | 16 (R) | 4 (R) | 8 (R) | 20 (S) |
| SM9 | 1331 | 128 (R) | 0.25 (S) | 64 (R) | 64 (R) | 16 (R) | 8 (I) | 1 (S) | 4 (R) | 4 (R) | 20 (S) |
| SM7 | 1332 | 128 (R) | 64 (R) | 64 (R) | 64 (R) | 16 (R) | 8 (I) | 4 (I) | 1 (S) | 1 (S) | 20 (S) |
| SM61 | 1333 | 128 (R) | 1 (S) | 64 (R) | 64 (R) | 16 (R) | 2 (S) | 1 (S) | 4 (R) | 4 (R) | 20 (S) |
| SM39 | 1335 | 64 (R) | 8 (I) | 8 (SDD) | 64 (R) | 16 (R) | 4 (S) | 16 (R) | 4 (R) | 2 (R) | 20 (S) |
| SM34 | 1336 | 128 (R) | 0.5 (S) | 64 (R) | 64 (R) | 16 (R) | 2 (S) | 1 (S) | 4 (R) | 4 (R) | 20 (S) |
| SM15 | 1337 | 128 (R) | 0.25 (S) | 8 (SDD) | 64 (R) | 8 (R) | 2 (S) | 1 (S) | 4 (R) | 8 (R) | 20 (S) |
| SM17 | Unknown | 128 (R) | 0.5 (S) | 64 (R) | 64 (R) | 16 (R) | 2 (S) | 1 (S) | 4 (R) | 8 (R) | 20 (S) |
| SM20 | Unknown | 128 (R) | 8 (I) | 64 (R) | 64 (R) | 16 (R) | 2 (S) | 1 (S) | 4 (R) | 4 (R) | 20 (S) |
| SM41 | Unknown | 64 (R) | 32 (R) | 8 (SDD) | 64 (R) | 16 (R) | 4 (S) | 16 (R) | 4 (R) | 4 (R) | 20 (S) |

Note: ST: sequence type; TZP: Piperacillin/Tazobactam; CAZ: Ceftazidime; FEP: Cefepime; ATM: Aztreonam; IPM: Imipenem; AK: Amikacin; CN10: Gentamicin; CIP: Ciprofloxacin; LEV: Levofloxacin; SXT: Trimethoprim/Sulfamethoxazole; S:Susceptible; I: Intermediate; R: Resistant; SDD: Susceptible-dose dependent;
