# Supplementary material for: Emergence of high-risk ST595 and ST640 clones of carbapenem-resistant Serratia marcescens: insights from genomic and virulence profiling during a nosocomial epidemic
Source: Front Microbiol. 2025 Oct 9;16:1681911. doi: 10.3389/fmicb.2025.1681911 (PMC12546152; doi:10.3389/fmicb.2025.1681911)
Supplement: Supplementary file 2 [file Data_Sheet_1.docx]

This Whole Genome Shotgun project has been deposited at DDBJ/ENA/GenBank

under the Bioproject ( PRJNA989674), the accession number were as follows:

JAVIOS000000000: Serratia marcescens SM1

JAVIOT000000000: Serratia marcescens SM10

JAVIOU000000000: Serratia marcescens SM11

JAVIOV000000000: Serratia marcescens SM12

JAVIOW000000000: Serratia marcescens SM15

JAVIOX000000000: Serratia marcescens SM17

JAVIOY000000000: Serratia marcescens SM18

JAVIOZ000000000: Serratia marcescens SM20

JAVIPA000000000: Serratia marcescens SM21

JAVIPB000000000: Serratia marcescens SM22

JAVIPC000000000: Serratia marcescens SM26

JAVIPD000000000: Serratia marcescens SM29

JAVIPE000000000: Serratia marcescens SM3

JAVIPF000000000: Serratia marcescens SM30

JAVIPG000000000: Serratia marcescens SM31

JAVIPH000000000: Serratia marcescens SM32

JAVIPI000000000: Serratia marcescens SM34

JAVIPJ000000000: Serratia marcescens SM35

JAVIPK000000000: Serratia marcescens SM36

JAVIPL000000000: Serratia marcescens SM37

JAVIPM000000000: Serratia marcescens SM38

JAVIPN000000000: Serratia marcescens SM39

JAVIPO000000000: Serratia marcescens SM4

JAVIPP000000000: Serratia marcescens SM40

JAVIPQ000000000: Serratia marcescens SM41

JAVIPR000000000: Serratia marcescens SM42

JAVIPS000000000: Serratia marcescens SM43

JAVIPT000000000: Serratia marcescens SM44

JAVIPU000000000: Serratia marcescens SM46

JAVIPV000000000: Serratia marcescens SM48

JAVIPW000000000: Serratia marcescens SM49

JAVIPX000000000: Serratia marcescens SM50

JAVIPY000000000: Serratia marcescens SM51

JAVIPZ000000000: Serratia marcescens SM52

JAVIQA000000000: Serratia marcescens SM53

JAVIQB000000000: Serratia marcescens SM54

JAVIQC000000000: Serratia marcescens SM55

JAVIQD000000000: Serratia marcescens SM56

JAVIQE000000000: Serratia marcescens SM57

JAVIQF000000000: Serratia marcescens SM58

JAVIQG000000000: Serratia marcescens SM59

JAVIQH000000000: Serratia marcescens SM60

JAVIQI000000000: Serratia marcescens SM61

JAVIQJ000000000: Serratia marcescens SM7

JAVIQK000000000: Serratia marcescens SM8

JAVIQL000000000: Serratia marcescens SM9

JAVIQM000000000: Serratia marcescens SM6

JAVIQN000000000: Serratia marcescens SM19

JAVIQO000000000: Serratia marcescens SM23

JAVIQP000000000: Serratia marcescens SM27

JAVIQQ000000000: Serratia marcescens SM45

JAVIQR000000000: Serratia marcescens SM47
